# Learning curve of semi-rigid ureteroscopy for small calculi: how many cases are necessary?

**DOI:** 10.1590/0100-6991e-20222693-en

**Published:** 2022-09-28

**Authors:** DANIEL ILIAS, CARLO CAMARGO PASSEROTTI, JOSÉ PONTES, FELIPE FAKHOURI, SABRINA THALITA DOS REIS FARIA, LINDA FERREIRA MAXIMIANO, JOSÉ PINHATA OTOCH, JOSE ARNALDO SHIOMI DA-CRUZ

**Affiliations:** 1 - Hospital Alemão Oswaldo Cruz,​ ​Centro Especializado em Urologia - São Paulo - SP - Brasil; 2 - Faculdade de Medicina da Universidade de São Paulo, Departamento de Técnica Cirúrgica e Cirurgia Experimental - São Paulo - SP - Brasil; 3 - Universidade de São Paulo, Hospital Universitário - São Paulo - SP - Brasil

**Keywords:** Learning Curve, Ureteroscopy, Ureteral Calculi, Urologic Surgical Procedures, Internship and Residency, Residência Médica, Ureteroscopia Semi-Rígida, Curva De Aprendizado, Ureterolitíase, Treinamento Cirúrgico

## Abstract

**Introduction:**

semi-rigid ureteroscopy is the procedure of choice for the treatment of ureterolithiasis, but it requires a learning curve to be performed safely.

**Objective:**

To describe an estimate of the learning curve for performing semi-rigid ureterorenolithotripsy in patients with small-sized ureterolithiasis and to estimate the minimum number of procedures necessary to safely perform the surgical procedure.

**Methods:**

this is a prospective study evaluating the learning curve of a resident of urology in the first 60 semirigid ureteroscopies in patients with ureterolithiasis up to 1cm. The patients were divided into three groups: Group I one to twenty surgeries, Group II twenty one to forty surgeries and Group III forty one to sixty surgeries. The surgeries were recorded and analyzed by two urologists experienced in endourology. A qualitative analysis was performed based on a previously validated tool and a quantitative analysis.

**Results:**

all qualitative variables had significant variation between Groups I and II (p<0.001), and between Groups I and III (p<0.001). There was a difference in time to access the ureter, passage of a double J catheter and total operative time between Groups I and II (p<0.001) and Groups I and III (p<0.001).

**Conclusion:**

after 40 cases there seems to be little increase in both quantitative as well as qualitative evaluation in surgical performance for performing semi-rigid ureterolithotripsy safely in calculations up to 1cm.

## RESUMO

**Introdução:**

ureteroscopia semi-rígida é o procedimento de escolha para o tratamento da ureterolitíase, mas necessita de uma curva de aprendizado para ser executada com segurança.

**Objetivo:**

descrever uma estimativa da curva de aprendizado para realização da ureterorrenolitotripsia semi-rígida em pacientes com ureterolitíase de pequena dimensão e estimar o número mínimo de procedimentos necessários para realizar o procedimento cirúrgico com segurança.

**Métodos:**

trata-se de um estudo prospectivo avaliando a curva de aprendizado de um residente de urologia nas primeiras 60 ureteroscopias semi-rígidas em pacientes com ureterolitíase até 1cm. Os pacientes foram divididos em três grupos: Grupo I uma a vinte cirurgias, Grupo II vinte e uma a quarenta cirurgias e Grupo III quarenta e uma a sessenta cirurgias. As cirurgias foram gravadas e analisadas por dois urologistas experientes em endourologia. Foi feita uma análise qualitativa baseada em uma ferramenta previamente validada e uma análise quantitativa.

**Resultados:**

todas as variáveis qualitativas tiveram variação significativa entre os Grupos I e II (p<0.001), e entre os Grupos I e III (p<0.001). Houve diferença no tempo para acesso ao ureter, passagem de cateter duplo J e tempo operatório total entre os Grupos I e II (p<0.001) e nos Grupos I e III (p<0.001).

**Conclusão:**

após 40 casos parece haver pouco incremento tanto na avaliação quantitativa bem como na avaliação qualitativa em performance cirúrgica para a realização de ureterolitotripsia semi-rígida com segurança em cálculos de até 1cm.

## INTRODUCTION

The first ureteroscopy was described in 1912 by Young and McKay, when a pediatric cystoscope was inadvertently inserted into the renal pelvis of a child with a dilated ureter, this finding being published in 1929^1^.

Ureteroscopy was introduced into clinical practice in the 1980s, when the first ureteroscope was produced by the urologist Perez-Castro in association with Karl Storz. The first semi-rigid ureteroscope was introduced into clinical practice in 1989, replacing the rigid model, as it allows flexion of the vertical axis without image distortion^1^ ^,^ ^2^.

It is a diagnostic and therapeutic procedure for urolithiasis, ureteral stenosis, and ureteral neoplasms^1^ ^,^ ^3^. Ureterolithiasis is the most common clinical condition requiring treatment with ureteroscopy^4^.

Ureterolithiasis can be treated with endoscopic surgery. The miniaturization of ureteroscopes associated with the introduction of the homium laser (Ho:YAG) improved stone-free rates and decreased complications resulting from the surgical procedure. Most calculi can be disintegrated with the laser and the holmium energy is completely absorbed by the water within five millimeters, ureter injuries being rare^5^ ^,^ ^6^.

The learning curve of semi-rigid ureteroscopy is not well described, especially for small calculi. The minimum number of cases to perform this procedure safely is still uncertain^4^ ^,^ ^7^ ^-^ ^11^.

## GOALS

To describe the endourology learning curve of semi-rigid ureteroscopy in patients with ureterolithiasis for calculi of up to 1cm and to estimate the minimum number of procedures necessary to safely perform this procedure.

## METHODS

This is a prospective study approved by the Ethics and Research Committee of our institution, carried out in a hospital with a urology residency program, where we evaluated the learning curve of a resident in his first 60 semi-rigid ureteroscopies in patients with ureterolithiasis. The surgeries were recorded and analyzed by two urologists experienced in endourology. We also performed: a qualitative analysis based on a tool previously published in the medical literature by Vassiliou et al (Table 1), which consists of five parameters: tissue management, bimanual dexterity, depth perception, autonomy, and efficiency; and a quantitative analysis, based on surgical times for accessing the ureter, calculi treatment, double-J catheter insertion, and surgical time^12^.

**Table 1:** Global rating scale of the intraoperative assessment tool.

Depth perception^a,b^
1. Constantly overshoots target, wide swings, slow to correct
2.
3. Some overshooting or missing of target but quick to correct
4.
5. Accurately directs instruments in the correct plane to the target
Bimanual dexterity^a,b^
1. Uses only one hand, ignores nondominant hand, poor coordination
2.
3. Uses both hands but does not optimize interaction between hands
4.
5. Expertly uses both hands in a complementary way to provide best exposure
Efficiency^a,b^
1. Inefficient efforts; many uncertain moves; constantly changing focus or persisting without progress
2.
3. Slow but planned moves; movements are reasonably organized
4.
5. Confident, efficient, and safe conduct; maintains focus on task, fluid progression
Handling of tissues^a,b^
1. Rough moves, tears tissue, injuries nearby structures, poor control, frequent suture breakage
2.
3. Handles tissue reasonably well, minor trauma to adjacent tissue (ie, occasional unnecessary bleeding or device slippage)
4.
5. Handles tissues well, applies adequate traction, negligible injury to adjacent structures
Autonomy^a, b^
1. Unable to complete the entire task, even with verbal guidance
2.
3. Able to complete task safely with moderate guidance
4.
5. Able to complete tasks independently without guidance.

^a^2= middle ground between degrees 1 and 3; ^b^4 = middle ground between degrees 3 and 5.

This study had the participation of 60 patients, 35 male and 25 female, and the surgeries performed were divided into three groups: from the first to the twentieth surgery (Group I), from the twenty-first to the fortieth procedure (Group II), and from the forty-first to sixtieth surgery (Group III).

Indications for semi-rigid ureteroscopy were ureterolithiasis with persistent pain, failure of clinical management with tamsulosin, and patient choice for surgical treatment. We excluded patients with ureterolithiasis associated with urinary tract infection and cases in which the calculus migrated to the kidney during intervention.

All procedures were performed with patients under spinal anesthesia, in lithotomy position. The ureteroscope was introduced through the urethra, a cystoscopy was performed to identify the ureteral meatus, a 0.035mmx150cm hydrophilic guide wire was inserted through the ureteral meatus towards the renal pelvis, and its placement was confirmed by radioscopy. Ureteroscopy was performed with calculus identification and a tipless nitinol calculus extractor was passed and positioned before the calculus to prevent migration to the kidney. The calculus was fragmented with a 200 micrometer laser fiber with a power of 10 watts, the residual fragments being removed with the calculus extractor. A 4.8 Fr x 20-26cm double J catheter was inserted at the end of the surgery. All procedures were performed with radioscopy and were recorded for further analysis. We collected demographic data and calculus size and location, and followed all patients for at least four weeks after double J catheter removal.

We evaluated the normally of continuous variables with the Kolmogorov-Smirnov test. Normally distributed variables were analyzed by two-tailed Student’s t test or ANOVA. After ANOVA, we performed the Tukey’s post-test for intergroup comparison. Variables that did not show a normal distribution were analyzed using the Mann-Whitney test. Qualitative variables were compared using the Mann-Whitney test. We analyzed categorical variables using the chi-square or Fisher’s exact test. The significance level in this study was 5%. The software used was StatPlus® v. 2009 for Mac.

## RESULTS

The mean age of patients was 39±13.7 years, with 35 (58.4%) males and 25 (41.6%) females. There was no statistical difference in the size, location, and laterality of the calculi between groups: 4.7±1.62 x 5.76±3.49 x 5.42±2.27 (p=0.37, groups I, II and III, respectively) (Table 2). All qualitative variables had significant variation between Groups I and II (p<0.001), as well as between Groups I and III (p<0.001). There was no statistical difference between Groups II and III in any qualitative variable (Table 3). There was no difference in time for calculus treatment between groups (p=0.14). There was a difference in the time for ureter access, double J catheter passage, and total operative time between Groups I and II (p<0.001), between Groups I and III (p<0.001), and there was no difference between groups II and III (Table 4). There were no intraoperative complications. Two patients in group I displayed intolerance to the double J catheter.

**Table 2:** Patients’ data.

Age (years±SD)	p=0.86
Global	39±13.7
Group I	40±9.5
Group II	39.45±17.5
Group III	37.7±13.6
Calculus dimension (mm±SD)	p=0.37
Global	5.3±2.5
Group I	4.7±1.62
Group II	5.7±3.49
Group III	5.42±2.27
Gender (male/female)	p=0.50
Global	35/25
Group I	8/12
Group II	7/13
Group III	10/10
Laterality (right/left)	p=0.41
Global	26/34
Group I	12/8
Group II	7/13
Group III	9/11
Complications	p>0.99
Global	2(3.3%)
Group I	2(10%)
Group II	0(0%)
Group III	0(0%)
Calculus location	p=0.61
Global	
Proximal ureter	6(10%)
Middle ureter	14(23%)
Distal ureter	40(67%)
Group I	
Proximal ureter	3(15%)
Middle ureter	7(35%)
Distal ureter	10(50%)
Group II	
Proximal ureter	1(5%)
Middle ureter	5(25%)
Distal ureter	14(70%)
Group III	
Proximal ureter	2(10%)
Middle ureter	5(25%)
Distal ureter	16(80%)

**Table 3:** Qualitative analysis.

	Handling of tissues	Bimanual dexterity	Autonomy	Depth perception	Efficiency
Group I (mean±SD)	3,45±0,9	3,25±1,0	3,65±0,9	3,4±0,7	3±0,7
Group II (mean±SD)	4,14±0,7	4,4±0,7	5±0	4,61±0,6	4,4±0,6
Group III (mean±SD)	4,6±0,4	4,7±0,4	4,9±0,2	4,85±0,3	4,7±0,3
p	p<0,001	p<0,001	p<0,001	p<0,001	p<0,001
Group I vs Group II (p)	<0,05	<0,01	<0,01	<0,01	<0,01
Group II vs Group III (p)	not significant	not significant	not significant	not significant	not significant

**Table 4:** Quantitative analysis.

	Access to the ureter (min)	Calculus treatment (min)	Passage of double J catheter (min)	Total operative time (min)
Group I (mean±SD)	8.25±5.2	11.2±12.1	4.45±1.9	25.65±17.2
Group II (mean±SD)	3.2±1.5	7.3±6.0	2.52±1.1	14.61±7.5
Group III (mean±SD)	3.23±1.7	6.1±5.1	2.23±1.4	13.23±6.2
p	p<0.001	p=0.14	p<0.001	p=0.001
Group I vs Group II (p)	<0.01	-	<0.01	<0.05
Group II vs Group III (p)	not significant	-	not significant	not significant

## DISCUSSION

In our study, we observed a statistically significant difference between groups I and II (p<0.001) in the qualitative analysis that assessed tissue handling, bimanual dexterity, autonomy, depth perception, and efficiency, but when comparing groups II and III, there is no such difference. In the quantitative analysis, which evaluates the time of access to the ureter, treatment of the calculus, passage of double catheter, and duration of surgery, we observed a statistically significant difference (p<0.001) in access to the ureter, of 8.25±5.2 x 3.2±1.5 minutes, passage of the double J catheter, of 4.45±1.9 x 2.52±1.1 minutes, and surgery duration, of 25.65±17.2 x 14.61±7.5 minutes between groups I and II, respectively. There was no significant difference in the quantitative analysis between groups II and III. When comparing patient age, sex, calculus size and location, laterality, and complications, we did not observe any statistical difference between the three groups.

Librenjak et al. retrospectively study 422 patients undergoing ureterorenoscopy at the Department of Urology of the Clinical Hospital Center in Split, Croatia, between 2001 and 2009. The surgical procedures were performed by eight urologists divided into two groups: first group with four urologists who had had endourology training since the beginning of their fourth-year specialization, and the second group with four urologists who started endourological procedures on average five years after the end of their specialization. They observed that the first group had higher success rates in treating middle and distal ureter calculi and removed larger calculi in the distal ureter^2^.

A retrospective study at the Department of Urology Hamad Medical Corporation in Doha, Qatar, from July 2008 to July 2011, carried out by Al-Naimi et al. involved 891 patients who underwent 1,182 ureteroscopies. Patients were divided into two groups, the first being operated by urology residents supervised by a urologist, and the second group formed by urologists with experience in endourology for two years after medical residency. The residents had a 90.3% calculus-free rate and a 10.5% complication rate, and the urologists, 91.1% and 13%, respectively^4^. This result shows that, depending on the practice and learning curve, there will be surgical success and complications rates very close to those of experienced professionals, as we found in our study.

Brunckhorst et al., in a cohort, randomized, controlled study involving 32 medical students from six UK universities, used the Delphi methodology and divided participants into two groups of 16 people, both receiving a didactic introduction to basic anatomy, familiarization with the equipment, and surgical steps. The procedure was performed on models, not patients, and the video recorded by the Uro-Scopic Trainer^TM^ simulator (Limbs & Things Ltd. Bristol, UK), a physical bench model, and the URO Mentor^TM^ (Simbionix, Cleveland, USA), a virtual reality simulator in the “Igloo” environment. Two blind specialists evaluated the videos, analyzing task completion time, ureter catheterization time, calculus removal, and stent installation. They used the objective structured assessment of surgical skills (OSATS) scale, which assesses seven aspects of global classification of technical skills, the rigid ureteroscopy evaluation score (RUES), and the non-technical skills for surgeons (NOTSS), which assesses four parameters: situational awareness, decision making, communication and teamwork, and leadership. The intervention cohort group (n=16) received ureteroscopy training and the non-control group (n=16) received no training. The group that received training was significantly faster and performed better than the control group on the OSATS and NOTSS scales^7^.

A multicenter study by Brehmer and Swartz evaluated performance before and after training in cystoscopy and semi-rigid ureteroscopy with 26 urology residents from Denmark and Norway divided into three groups and evaluated by an experienced urologist using the OSATS test, finding a significantly better performance in all groups after endourological training^8^. 

Currently, there are few prospective studies addressing the learning curve in endourological calculus treatment procedures and none focused on the learning curve for semi-rigid ureteroscopy^13^.

The main limitation in our work is the number of surgeons evaluated. On the other hand, several previously published articles evaluated the learning curve of a single surgeon, with very valuable and interesting results^14^ ^-^ ^16^.

Studies on the learning curve are extremely important to design the training of resident physicians in residency and fellowship programs to safely perform surgical procedures and better train surgeons.

## CONCLUSION

After 40 cases, there seems to be little increase in both the quantitative and qualitative assessments of surgical performance. Thus, 40 cases seem to be enough for a surgeon to safely perform a semi-rigid endoscopic ureterolithotripsy on calculi of up to 1cm.
